# Optimal time of starting tocilizumab in acute phase of adult-onset Still’s disease and comparison of its efficacy with that of methotrexate: a case series and a review of the literature

**DOI:** 10.1007/s10067-024-06905-8

**Published:** 2024-02-12

**Authors:** Satoshi Suzuki, Yuko Kataoka, Tomoya Otani, Yukino Taniguchi, Keigo Ikeda, Naoto Tamura, Shinji Morimoto

**Affiliations:** 1 Department of Internal Medicine and Rheumatology, Juntendo University Urayasu Hospital, Chiba, Japan; 2 Department of Internal Medicine and Rheumatology, Juntendo University School of Medicine, Tokyo, Japan

**Keywords:** Adult-onset still’s disease, Glucocorticoids, Methotrexate, Tocilizumab

## Abstract

Adult-onset still’s disease is a rare condition that is generally treated by glucocorticoids. Importantly, due to the limited established treatments, glucocorticoid-refractory cases are particularly difficult to treat. Between December 2009 and August 2022, nine patients with adult-onset Still’s disease were treated with tocilizumab (tocilizumab group). The therapeutic efficacy and safety of tocilizumab initiation in the acute phase were evaluated in cases of initial onset and recurrence. We also compared the efficacy of tocilizumab with that of methotrexate (methotrexate group, *n* = 13), which has been the drug of choice for adjunctive therapy. Tocilizumab demonstrated the expected efficacy in all four patients who received it at relapse and in three of the five patients who received it at the initial onset. However, two patients developed macrophage activation syndrome following treatment. A comparison of treatment effects between the methotrexate and tocilizumab groups revealed that the ferritin and C-reactive protein levels, severity score, and glucocorticoid doses decreased over time in both groups; nonetheless, the tocilizumab group experienced a more stable effect. Tocilizumab is undoubtedly a valuable treatment option for adult-onset Still’s disease, especially when administered at relapse. This suggests that it shows both high safety and good efficacy. Nevertheless, a larger sample size is required to validate the efficacy and safety of tocilizumab compared with those of the existing alternatives.

**Table:** 

**Key Points**
**• ***We examined the significance of TCZ in terms of therapeutic efficacy, reduction in glucocorticoid usage, and safety in patients with AOSD*.
**•** *We compared the therapeutic efficacy of TCZ with that of MTX, which is often used to treat glucocorticoid-resistant AOSD*.
**•** *TCZ is undoubtedly a valuable treatment option for AOSD, especially when administered at relapse, suggesting both high safety and good efficacy*.

## Introduction

Adult-onset still’s disease (AOSD) is a systemic inflammatory disease that was first described by Bywaters in 1971 [1]; it is characterized by its similarity to still’s disease, a pediatric disease first reported by the British pediatrician George Frederic Still in 1896. Still’s disease is more commonly referred to as juvenile idiopathic arthritis (JIA) [2], and to date, AOSD remains the only subtype. Patients with the disease typically present with nonspecific symptoms, such as fever, sore throat, arthralgia, and skin rashes. In the absence of specific autoantibodies or imaging findings, diagnosis requires the exclusion of infectious diseases, malignancy, and rheumatic diseases other than AOSD.

The prognosis of AOSD is generally good, but some patients may develop fatal hemophagocytic syndrome (HPS) or disseminated intravascular coagulation syndrome. However, treatment options are largely limited to glucocorticoids, and the treatment of severe cases can be difficult. In this context, an additional indication for the biological anti-rheumatic drug tocilizumab (TCZ) for AOSD was approved by the Japanese health insurance authority in 2019; the subsequent addition of TCZ to the treatment options for AOSD has had a significant impact. However, a consensus is lacking on the optimal timing for introducing TCZ, specifically, whether it should be used aggressively at the initial onset or upon recurrence despite continued conventional therapy. Therefore, this study focused on patients with AOSD who were hospitalized and treated with TCZ, aiming to examine the significance of TCZ in terms of therapeutic efficacy, reduction in glucocorticoid usage, and safety. We also compared the therapeutic efficacy of TCZ with that of methotrexate (MTX), which is often used to treat glucocorticoid-resistant AOSD.

## Materials and methods

### Patients

A total of 43 patients with AOSD were admitted to the Juntendo University Urayasu Hospital between December 2009 and August 2022; among them, we included nine patients who received TCZ (TCZ group) and 13 patients who received MTX (MTX group; patients with no history of TCZ use). All patients with AOSD were diagnosed based on Yamaguchi’s classification criteria, which have shown high sensitivity and specificity for AOSD [3, 4]. The laboratory and physical findings of the TCZ group at the time of TCZ introduction are shown in Table 1. Informed consent was obtained via an opt-out manner from the institute’s website in accordance with the guidelines of the Ethics Committee of Juntendo University (approval no. E23-0219).

**Table 1:** Laboratory and physical findings at the initiation of tocilizumab

	Cases of relapse				Cases of initial onset				
	Case.1	Case.2	Case.3	Case.4	Case.5	Case.6	Case.7	Case.8	Case.9
Age (y.o) / Gender (F/M)	72/F	66/F	46/F	33/F	69/F	44/M	50/F	58/F	55/M
Height (cm) / Body Weight (kg)	148/36	148/42	157/52	155/41	151/38	164/61	162/55	167/59	174/58
Disease duration (months)	67	13	26	31	−	−	−	−	−
Time from onset to TCZ induction (months)	−	−	−	−	3 weeks	1	2	3	1
Time from relapse to TCZ induction (months)	3	25 days	3	2	−	−	−	−	−
Dose of PSL (mg/day)	30	40	15	40	45	55	50	60	65
Fever	−	−	−	−	−	−	+	** + **	−
Sore throat	−	−	−	−	−	−	−	−	−
Rush	−	+	−	+	+	−	+	+	+
Arthritis	+	−	+	+	−	+	+	+	−
Lymphadenopathy	−	−	−	−	−	−	+	−	−
Hepatomegaly / Splenomegaly	−	−	+	+	−	+	+	+	+
White blood cell (/μl)	7300	5200	9400	34100	17200	20700	8100	9700	15700
Neutrophil (/μl)	4891	3692	8902	29497	15772	13726	4609	7478	14302
Lymphocyte (/μl)	1868	1092	150.4	2728	774	2546	2705	1552	518.1
Monocyte (/μl)	365	270	244	1535	34	476	502	563	753.6
Hemoglobin (g/dl)	8.9	13.4	11.1	14	10	11	10.4	10.8	15.6
Ferritin (ng/ml)	113	910	300	649	9459	3177	540	6035	2250
AST/ALT (IU/l)	22/28	20/22	33/58	69/118	63/126	31/94	8/11	15/33	32/109
LDH (IU/l)	249	290	225	234	542	139	137	250	159
CRP (mg/dl)	3.1	0.3	9.6	2.7	7.2	5.6	2.6	1.3	1.6
sIL-2R (U/ml)	945	N/A	N/A	1150	1380	2260	771	851	993
IL-6 (pg/ml)	N/A	N/A	N/A	N/A	N/A	111	N/A	N/A	383
IL-18 (pg/ml)	N/A	N/A	N/A	N/A	N/A	> 5000	> 5000	> 5000	N/A

AST: aspartate transaminase; ALT: alanine transaminase; LDH: lactate dehydrogenase; CRP: C-reactive protein; IL: interleukin

### Methods

All patients in the TCZ group were administered TCZ due to their resistance to conventional glucocorticoid therapy; they received 8 mg/kg of TCZ intravenously every 2 weeks. The TCZ group was further divided into two subgroups: the initial induction (patients who received TCZ at the initial onset [*n* = 5]) and recurrent induction (patients who received TCZ at relapse [*n* = 4]) groups. Patients in the MTX group received MTX orally at the maximum tolerable dose of up to 16 mg/week, which is the approved dosage for treating rheumatoid arthritis in Japan. The MTX and TCZ groups were compared in terms of the changes in the ferritin levels, C-reactive protein (CRP) levels, modified Pouchot score (Rau score) -AOSD severity indicators, and glucocorticoid doses from initiation to 2, 4, 12, and 24 weeks thereafter. Additionally, we analyzed the test results of routine assessments performed during general examinations at the Juntendo University Urayasu Hospital. Patients who received both TCZ and MTX were excluded from the comparisons. In this study, relapse was defined as episodes requiring rehospitalization for intensified treatment of AOSD. The Wilcoxon signed-rank test was used to analyze the effects of TCZ treatment after initiation. We used the χ^2^-test to analyze and compare the background characteristics of the MTX and TCZ groups; *P* < 0.05 indicated statistical significance.

## Results

**Fig. 1:**
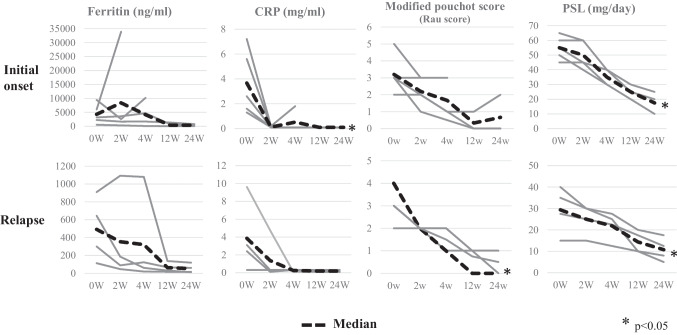
Effects of treatment after introduction of TCZ. TCZ initiation in the acute phase was effective both at the initial onset and at relapse; however, patients in cases #5 and #8, in whom the ferritin levels increased during the course of the disease despite TCZ administration, developed the macrophage activation syndrome. TCZ, tocilizumab

**Fig. 2:**
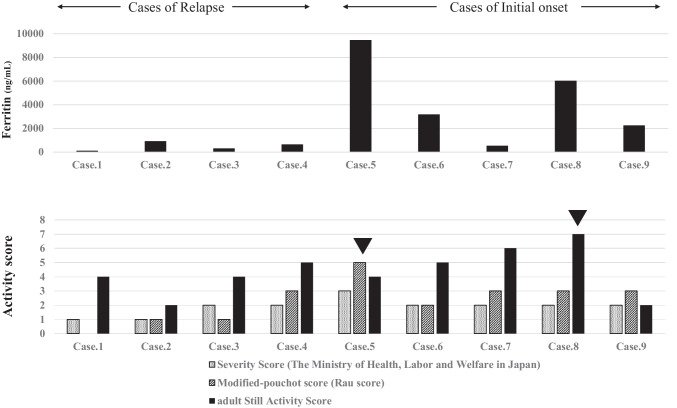
The ferritin levels were considerably higher in patients in cases #5 and #8 (who presented with severe disease) than in the other patients at TCZ initiation; the modified Pouchot scores (Rau scores) and adult Still Activity Scores were also higher in these two patients (indicated by filled triangles). TCZ, tocilizumab

**Fig. 3:**
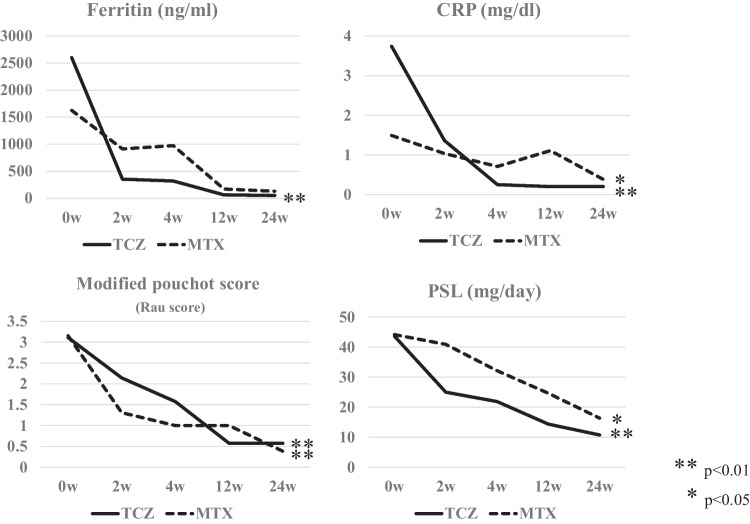
Comparison of treatment effects between the MTX and TCZ groups. The ferritin and CRP levels and the glucocorticoid dose decreased over time in both groups; however, the TCZ group is seen to experience a more favorable effect. MTX, methotrexate; TCZ, tocilizumab

Compared with the recurrent induction group, the initial induction group tended to show higher glucocorticoid usage at TCZ initiation and had higher ferritin and CRP levels as well as higher white blood cell counts (Table 1). The ferritin and CRP levels modified the Pouchot score (Rau score), and glucocorticoid usage decreased over time in both the initial and recurrent induction groups. However, in two patients in the initial induction group (cases #5 and #8), the ferritin level continued to increase during the course of the disease despite TCZ use; both patients developed macrophage activation syndrome (MAS) and died (Fig. 1). The patient in case #5 was diagnosed with MAS based on bone-marrow biopsy findings; conversely, the patient in case #8 was diagnosed with MAS based on autopsy findings (which confirmed HPS) and findings of fever and a decreased blood count [5]. Compared with the other patients, these two patients had a more severe disease and considerably higher ferritin levels at TCZ initiation; their modified Pouchot scores (Rau scores) [6] and adult Still Activity Scores (SASs) [7] were also higher (Fig. 2). The Severity Score (Ministry of Health, Labor and Welfare in Japan) was developed based on the results from a nationwide epidemiological survey of AOSD conducted in Japan in 2010 [8, 9]; it is used to determine public subsidies. The SASs did not differ significantly between the patients with MAS and the other patients. A comparison of treatment effects between the MTX and TCZ groups revealed that the ferritin level, CRP level, modified Pouchot score (Rau score), and glucocorticoid doses decreased over time in both groups; however, a more favorable effect was noted in the TCZ group than in the MTX group (Fig. 3). The background characteristics of the TCZ and MTX groups are shown in Table 2.

**Table 2:** Background characteristics of the MTX and TCZ groups

	TCZ (*n* = 9)	MTX (*n* = 13)	*P*-value
Age (y.o)	55 ± 13	43 ± 12	.0429
Female (n)	7	10	/
New onset (n)	5	10	/
Disease duration (months)	17 ± 23	8.5 ± 16	.3735
Dose of PSL (mg/day)	44 ± 16	44 ± 16	.9678
Dose of MTX (mg/week)	/	5.2 ± 1.5	/
Dose of TCZ (mg)	373 ± 56	/	/
ferritin (ng/ml)	2583 ± 3209	1033 ± 1126	.1977
CRP (mg/dl)	3.8 ± 3.0	1.5 ± 1.9	.0712
WBC (/μl)	14155 ± 9073	11546 ± 4646	.4441
AST (IU/l)	64 ± 98	23 ± 10	.2369
ALT (IU/l)	67 ± 45	32 ± 23	.0634
LDH (IU/l)	247 ± 123	262 ± 115	.7764

MTX: methotrexate; TCZ: tocilizumab

## Discussion

AOSD is a benign disease with a generally good prognosis; however, it is rare, and no treatment other than glucocorticoids has been established to date. Therefore, reducing the glucocorticoid dose is difficult in severe cases or in cases of recurrences; consequently, drug-related adverse events (including infections) are often problematic. Although the combination of MTX (a disease-modifying anti-rheumatic drug) and cyclosporine (an immunosuppressive drug) has been used as a treatment for a while, the onset of its therapeutic effect varies greatly from one case to another; furthermore, these drugs have not yet gained full recognition as treatments for AOSD [10–12]. Against this backdrop, the effectiveness of TCZ, a biological anti-rheumatic drug, was reported in a clinical study on 27 patients with glucocorticoid-resistant AOSD in Japan [13].

Subsequently, an additional indication was approved by the Japanese Health Insurance for AOSD in 2019. TCZ is the first biological anti-rheumatic drug recommended for AOSD treatment in Japan; this addition to the available treatment options for AOSD has had a considerable effect on AOSD management. TCZ is an anti-interleukin (IL)-6 receptor antibody that inhibits both soluble and membrane-bound IL-6 receptors, thereby reducing IL-6 activity in vivo. IL-6, initially isolated in 1986 as a soluble factor from T cells that stimulates B cells to differentiate into antibody-producing cells, plays a role in the pathogenesis of various diseases, such as autoimmune diseases, cancers, and osteoporosis [14, 15]. Notably, TCZ has already been used for treating JIA both in Japan and overseas [16–18]. Even before its official approval for AOSD, it was reported to reduce the relapse rates in AOSD [19] and, in comparison with infliximab, had higher rates of treatment continuation [20].

In the present study, TCZ effectively reduced the activity of acute AOSD in both the relapsed and first-episode groups. However, MAS development has been reported in several cases [21, 22], and caution should be exercised when administering TCZ. Yamabe et al. reported secondary MAS in six of their 20 patients with AOSD who received TCZ [23], suggesting that MAS after TCZ use may be more common than expected. The etiology of MAS after TCZ administration remains unclear; however, it may be because TCZ is an IL-6 receptor antibody, rather than a drug that directly neutralizes IL-6, and serum IL-6 levels temporarily increase after TCZ administration [24]. In our study, two of the five patients who received TCZ at the initial onset developed MAS; conversely, all four patients who received TCZ at relapse did well, with no dropouts until the time of writing. Therefore, TCZ may be safer for the induction of remission at relapse than at the initial onset.

The initiation of TCZ may be risky in cases showing uncontrolled disease activity, such as in those with high ferritin levels, despite the use of high-dose glucocorticoids. Although AOSD differs from rheumatoid arthritis and systemic lupus erythematosus in that there are very few established indices of disease activity, the patients in our study who developed MAS after TCZ initiation had higher modified Pouchot scores (Rau scores) and SASs; in particular, the SAS was the highest among patients with MAS (maximum score: 7). The SAS is a new activity score developed based on the findings from 197 patients with AOSD in 2022 and encompasses the following four components: fever, arthralgia, neutrophilia, and ferritin elevation. It is characterized by its emphasis on fever and arthralgia, particularly frequent in AOSD, and by its consideration of elevated ferritin levels [7]. In addition, the patient in case #5 had a history of cytomegalovirus infection and *Pneumocystis* pneumonia after TCZ initiation and was treated with valganciclovir and sulfamethoxazole/trimethoprim; therefore, either the infection or the drugs could have induced MAS. Thus, patients with AOSD who develop infections or require multiple medications other than glucocorticoids after starting treatment are considered at risk for MAS and should not undergo prompt TCZ initiation. Nevertheless, it was difficult to predict MAS development before TCZ initiation using a single index; as more cases accumulate, risk assessment by combining multiple indexes may become possible. Although studies have compared anti-IL-1 and immunosuppressive agents [25], none have compared TCZ and MTX. In our study, both the TCZ and MTX groups showed reductions in the ferritin and CRP levels and in the glucocorticoid doses over time; however, the TCZ group experienced a more favorable effect.

This study had several limitations. First, this was a single-center study, and the number of patients was small because of the rarity of the disease. Moreover, the patient background characteristics could not be standardized for the comparison of treatment effects, and minor flares that did not require hospitalization were excluded.

In conclusion, initiating TCZ treatment during the acute phase has proven effective, both at the initial onset and during relapse. However, it is essential to note the potential of MAS after cytokine inhibition therapy, as reported previously. Despite the absence of a clear mechanism or causal relationship, TCZ is undoubtedly a valuable treatment option for AOSD, especially when administered at relapse. Comparing TCZ and MTX, both demonstrated similar effectiveness in the acute phase of AOSD; however, the TCZ treatment group demonstrated more favorable results. Although the study had a limited sample size, it represents the first direct comparison between TCZ and MTX. We believe that our findings may serve as a basis for establishing safer and more reliable treatment methods for AOSD in the future.

## Data Availability

The data underlying this article will be shared upon reasonable request to the corresponding author.
